# Bupivacaine-Loaded PLGA Nanoparticles for Long-Term Management of Chronic Neuropathic Pain

**DOI:** 10.3390/pharmaceutics18101226

**Published:** 2026-09-27

**Authors:** Tao Wang, Keyun Tang, Fengrun Sun, Yujia Ma, Yehong Fang, Ning Yu, Fan Liu, Chao Ma

**Affiliations:** 1 Department of Human Anatomy, Histology and Embryology, State Key Laboratory of Common Mechanism Research for Major Diseases, Neuroscience Center, Joint Laboratory of Anesthesia and Pain, Institute of Basic Medical Sciences Chinese Academy of Medical Sciences, School of Basic Medicine Peking, Union Medical College, Beijing 100005, China; fireyafireya@outlook.com (F.S.); 15210236312@163.com (Y.M.); fyhpumc@163.com (Y.F.); yuning97@126.com (N.Y.); machao@ibms.cams.cn (C.M.); 2 Department of Dermatology, State Key Laboratory of Complex Severe and Rare Diseases, Peking Union Medical College Hospital, Chinese Academy of Medical Sciences and Peking Union Medical College, National Clinical Research Center for Dermatologic and Immunologic Diseases, Beijing 100730, China; tky15@tsinghua.org.cn; 3 National Human Brain Bank for Development and Function, Beijing 100005, China; 4 Chinese Institute for Brain Research, Beijing 102206, China

**Keywords:** bupivacaine, nanoparticles, neuropathic pain, mouse, rat, CCD, CCI

## Abstract

**Background/Objectives:** Chronic neuropathic pain imposes a substantial burden on patients and society, and opioids are unsuitable for long-term use due to their addictive potential. To overcome this limitation, we encapsulated bupivacaine in PLGA nanoparticles to achieve sustained-release analgesia with reduced systemic toxicity. **Methods:** The analgesic efficacy was evaluated in rat models of chronic compression of the dorsal root ganglion (CCD) and chronic constriction injury (CCI). Nanoparticles were characterized using scanning electron microscopy (SEM) for morphology and size, and X-ray diffraction (XRD) for dispersion. In vivo electrophysiological recordings, including action potentials from DRG neuronal somata and compound action potentials in the sciatic nerve, were performed to investigate peripheral analgesic mechanisms, while plasma bupivacaine concentrations were measured by high-performance liquid chromatography (HPLC). **Results:** Bupivacaine nanoparticles significantly alleviated mechanical pain in both CCD and CCI rats, but had no effect on thermal pain. They selectively inhibited C-fiber conduction without affecting A-fibers. Importantly, plasma bupivacaine levels remained well below the corresponding toxic thresholds, indicating a favorable safety profile. **Conclusions:** These findings suggest that bupivacaine-loaded PLGA nanoparticles represent a promising long-term therapeutic option for chronic neuropathic pain management.

## 1. Introduction

Pain management remains a critical component of perioperative patient care. Even though opioid analgesics appear highly effective in postoperative analgesia, opioid-related adverse events, which exact a high toll on patient morbidity and total hospitalization consumption, generally limit the distribution of clinical analgesia [1]. Thus, multimodal analgesia regimens have been advocated for management of postoperative pain to reduce the requirement for opioid analgesics, and local anesthetics (e.g., bupivacaine, lidocaine) have increasingly become a mainstay of such approaches [2]. Increasing evidence supports that nanotechnology can serve as a valuable tool—both as drug delivery systems (including liposomes) and as nanocapacitor-based devices/patches—to reduce or even eliminate opioid use in surgical patients. However, further studies are required to validate this concept, especially with respect to the integration of nanotechnology into devices/patches [3].

However, single-dose injections of local anesthetics provide only a relatively short duration of action (typically 6–8 h) for peripheral nerve blocks [4]. Catheter-based delivery of local anesthetics carries risks of catheter dislodgement and irreversible tissue damage [5,6]. Therefore, numerous studies have focused on developing extended-release formulations to prolong the effects of local anesthetics. Various nanoparticle formulations have been assessed in vivo for neuropathic pain, including lipid nanoparticles, polymeric nanoparticles (e.g., PLGA nanoformulation and hybrid polymer-based nanoformulations), metal nanoparticles and silica nanoparticles [7]. Exparrel^®^, for example, a liposome formulation of 1.3% bupivacaine, is the only extended-release bupivacaine product approved by the U.S. Food and Drug Administration (FDA) [8]. While it effectively prolongs analgesia, its functional duration does not exceed 72 h, which may be insufficient to cover the entire postoperative recovery period [9]. Furthermore, the inherent instability of liposome can lead to burst release of bupivacaine and associated multi-organ toxicity, raising concerns about its clinical applicability [10]. A protein hydrogel incorporating bupivacaine nanoparticles achieved a 39.9 h nerve block in rats (versus 5.5 h for EXPAREL^®^), with benign tissue responses and sustained in vitro release [11]. Bupivacaine-encapsulated PLGA nanoparticles represent a novel extended-release formulation in which bupivacaine is embedded in a poly(lactide-co-glycolide) matrix [12]. Previous studies have shown that CCD (chronic compression of the dorsal root ganglion) alone or CCD combined with blank nanoparticles without bupivacaine induced persistent allodynia and hyperalgesia in the ipsilateral plantar hind paw of rodents [13,14,15]. We previously found that bupivacaine-loaded nanoparticles successfully prevented the occurrence of mechanical allodynia and hyperalgesia in established CCD models, with sustained in vitro release exceeding 35 days [13]. The present study was designed to evaluate the analgesic effects of these nanoparticles via behavioral testing and electrophysiological recordings in rodent (mouse and rat) models of nerve injury. Additionally, we explored the pharmacokinetic profile by measuring plasma bupivacaine concentrations in rats and assessed local tissue reactions via histopathological staining after direct injection of bupivacaine nanoparticle suspension into the L3 and L4 DRG and into the sciatic nerve compression in CCI rats.

## 2. Methods

### 2.1. Preparation of PLGA–Bupivacaine Nanoparticles

Bupivacaine-loaded nanoparticles were synthesized using a single-emulsion solvent evaporation technique as previously described [16]. Briefly, 100 mg of PLGA (50:50; Polysciences, Birmingham, AL, USA) and 40 mg bupivacaine were dissolved in 2 mL solvent of dichloromethane (DCM). The polymer solution was added dropwise to 4 mL of 2.5% polyvinyl alcohol (PVA). To allow solvent evaporation and particle hardening, the emulsion was sonicated 3 times in an iced bath (10 s each), then decanted into a beaker containing 0.3% (*v*/*v*) PVA, and stirred for 3 h at room temperature. The solution was centrifuged at 18,000 rpm for 30 min and the deposited particles were collected. Trehalose was added at a ratio of 0.5:1 (trehalose:nanoparticles) to reduce nanoparticle aggregation before lyophilization. Finally, an aqueous mixture of nanoparticles and sugar was prepared at a concentration of 60 mg/mL nanoparticles for injection.

### 2.2. Characterization of Bupivacaine-Loaded PLGA Nanoparticles

Nanoparticle morphology was examined using scanning electron microscopy (ZEISS MERLIN Compact, Carl Zeiss Microscopy GmbH, Oberkochen, Germany). Accelerating voltage was 15 kV. In X-ray diffraction (XRD) analysis, PLGA crystals and bupivacaine powders were placed on a glass slide separately. We used an X-ray diffractometer (D/max 2500 V, Rigaku Corporation, Akishima, Tokyo, Japan) with Cu Kα radiation (40 kV, 150 mA) to examine the diffraction spectra. The diffraction scans were recorded at 2θ values in the angular range of 5–60° with a step size of 0.02°.

The particle size, polydispersity index (PDI), and surface charge of PLGA–bupivacaine nanoparticles (PLGA–BUP NPs) were characterized by dynamic light scattering (DLS) and electrophoretic light scattering using a Zetasizer Nano ZS (Malvern Panalytical Ltd., Malvern, UK) at 25 °C. Freshly prepared NP suspensions were diluted to an appropriate concentration (approximately 0.1 mg/mL) with ultrapure water (pH 7.4), equilibrated for 120 s, and measured in triplicate. The Z-average diameter, PDI, and zeta potential were recorded. For FT-IR analysis, lyophilized PLGA–BUP NPs, blank PLGA NPs, free bupivacaine, PLGA, and their physical mixture were examined using an FT-IR spectrometer (Thermo Nicolet iS50, Thermo Fisher Scientific, Madison, WI, USA) equipped with an attenuated total reflectance (ATR) accessory. Spectra were collected over the range of 4000–400 cm^−1^ at a resolution of 4 cm^−1^ with 64 co-added scans, and background correction was performed. For ^1^H NMR analysis, lyophilized samples were dissolved in CDCl_3_ (5–10 mg/mL) with tetramethylsilane (TMS) as the internal standard. ^1^H NMR spectra were recorded on a 400 MHz superconducting magnet NMR spectrometer at 25 °C using a 30° pulse, a relaxation delay of 1–5 s, and 16–64 scans. Chemical shifts (δ) were reported in ppm, and spectra were processed using MestReNova software (Mnova 17.0.1). These characterizations were used to confirm the chemical structure, successful encapsulation, and possible drug–polymer interactions. DLS and zeta potential measurements were performed on three independently prepared batches, and data are presented as mean ± standard deviation (SD).

### 2.3. Animal Treatment

C57BL/6J male mice (body weight 25–35 g) and adult female Sprague–Dawley rats (body weight 150–160 g) were obtained from [Beijing, China]. Health and immune status were verified by the supplier’s health monitoring reports. All animals were housed in groups of 3–5 per cage with free access to rat chow and water. The room was maintained constantly at 23 ± 2 °C with a 12 h light/12 h dark diurnal cycle being preserved. All experiments were approved by the Institutional Animal Care and Use Committee of the Chinese Academy of Medical Sciences, Institute of Basic Medical Sciences (approval number: #211-2014). A random sequence was generated using the RAND function in Excel. To reduce confounding, the order of drug administration and the sequence of behavioral tests were randomized across animals. However, due to practical constraints, cage location was not randomized; all cages were kept on the same rack in a climate-controlled room, and we consider the environmental variation to be negligible. The timing of measurements was standardized (all tests performed between 10:00 and 12:00) to minimize diurnal effects. The experimenter was not blinded during the procedure, but the outcome assessor was blinded to group allocation. Humane endpoints were predefined: (1) >20% weight loss within 48 h; (2) inability to eat or drink for >24 h; (3) severe distress (self-mutilation, vocalization, immobility); (4) loss of righting reflex for >15 s. Animals were monitored twice daily. Any animal reaching an endpoint was immediately euthanized. No animal reached an endpoint before study completion.

### 2.4. CCD Surgery

Under 3–4% isoflurane, a 2–3 cm incision was made along the dorsal midline. The paraspinal muscles were separated by sharp and blunt dissection to expose the lateral aspects of L3 and L4 vertebrae. The lateral processes of vertebrae were clipped to allow injection into the dorsal root ganglia (DRG). A CCD model was produced by inserting an L-shaped stainless steel rod (0.3 mm diameter, each arm 2 mm in length) into the right intervertebral foramen to compress the DRG [13].Using a specific conducted micropipette [12], we carefully punctuated the exposed epineurium of the ganglion under a dissecting microscope; the pipette tip was advanced until it contacted the epineurium over L3 or L4 DRG. A 6 μL suspension was injected over 1–2 min for each site of ganglions. One group of rats received bupivacaine nanoparticle solutions (*n* = 5) while the other was injected with blank nanoparticles (*n* = 5). The overlying muscle and skin were closed in layers using monofilament nylon sutures. An ointment containing antibiotic, local anesthetic, and anti-inflammatory agents was applied to the incision. Systemic antibiotic (Baytril, 10 mg/mL, i.m.) was also administered.

### 2.5. CCI Surgery

In accordance with the study of Bennett and Xie [17], we performed chronic constriction injury (CCI) in the rats (*n* = 6) anesthetized with intraperitoneal injection of sodium pentobarbital (40 mg/kg) under aseptic conditions. After exposing the right sciatic nerve at mid-thigh level, four loose ligatures of chromic gut suture were tied around the nerve with approximately 1 mm spacing between knots. The sciatic nerves of sham animals were exposed but not ligated.

### 2.6. Behavior Tests

Withdrawal responses to mechanical stimuli were measured through electronic von Frey filaments one day before surgery to establish baseline values, and on days 1, 4, 7 and 14 after CCD surgery. Rats were placed in separate plastic chambers (7 × 20 × 20 cm) on an elevated stainless steel mesh floor 30 min before testing for acclimation. Rats were habituated to the test chamber to avoid any stimulation from anxiety or stress. The von Frey filament was applied vertically for 1 s, every 45–50 s, 10 times, to the plantar surface of each hand paw. A qualified response was defined as brisk withdrawal without vocalization. Thresholds were automatically recorded in grams. A 5 min interval was allowed between tests to ensure resting state. Mechanical hyperalgesia was defined as a decrease in the paw withdrawal threshold [17]. Using the Hargreaves method, animals were placed on a transparent glass plate; a mobile infrared heat source was focused onto the plantar surface of the hind paw. The apparatus automatically recorded the latency from heat stimulus onset to paw withdrawal response. Allocation was performed by an independent researcher not involved in subsequent stages. The experimenter, outcome assessor, and data analyst were all blinded to group allocation throughout the study. Blinding was maintained until final analysis. 

### 2.7. Electrical Recordings of Compound Action Potential (CAP) in Sciatic Nerve

To examine whether bupivacaine nanoparticles act on periphery analgesia by blocking axonal transduction of stimuli inputs, we recorded the active potential firings from the sciatic nerve after distal application of a sensitizing agent. Detailed procedures were presented in previous studies [18,19]. Briefly, under isoflurane anesthesia, the sciatic nerves of rats undergoing surgical treatment were exposed at mid-thigh, embedded in a pool of mineral oil and kept constantly at 35 ± 1 °C. For recording of CAP, a pair of stimulating electrodes was placed on the distal side of the sciatic nerve, and a recoding electrode was placed at 20 mm proximally. CAPs were evoked by repetitive electrical stimulation (0.2 Hz square-wave pulses, 0.5 ms duration, intensity up to 10 mA) and recorded using a custom low-noise differential amplifier and a Micro1401 interface with Spike6 software (Cambridge Electronic Design, Inc., Cambridge, UK). CAP waveforms were classified as C- (<1.5 m/s), Aδ- (1.5~15 m/s), or Aβ-component (>15 m/s) based on conduction velocity (CV) of impulses evoked by electrical stimulation to the sciatic nerve.

### 2.8. In Vivo Electrophysiological Recording Procedures

Recordings were performed in CCD mice 5–7 days after rod implantation. Under isoflurane anesthesia (1–2%, via tracheostomy, maintained with a SomnoSuite system (Kent Scientific Corporation, Torrington, CT, USA)), a laminectomy was performed at L3. The L3 DRG was fixed to a platform and superfused with warm artificial cerebrospinal fluid (ACSF) within a skin–ring pool. After removing the epineurium, neuronal somata on the DRG surface were visualized at 40× magnification under reflection microscopy. Collagenase P (1 mg/mL, 5 min) was applied locally to loosen cell bodies. A single soma was drawn into a glass micropipette (tip 20–25 μm, filled with ACSF), and action potentials (APs) were recorded extracellularly using a Multiclamp 700B amplifier. Spontaneous (ongoing) discharge was defined as APs persisting for >3 min without external stimulation, excluding physiologically normal endings (e.g., cool-sensitive thermoreceptors) or transient electrode artifacts.

Cutaneous receptive fields were mapped on the hind limb by gentle touch, pinch, a blunt glass probe, and von Frey filaments. A series of five von Frey filaments (tip diameter 200 μm; forces 2, 6, 20, 40, and 80 mN) were applied for 1 s each in ascending order. Finally, axonal conduction velocity was measured by electrically stimulating the receptive field with two wire electrodes and dividing the evoked AP latency by the distance between the field and the soma.

### 2.9. Immunohistochemistry and Microscopy

Fifteen days after CCD surgery, mouse L3/L4 DRG were collected following transcardial perfusion with PBS and 4% paraformaldehyde, post-fixed for 4 h, and cryoprotected in 30% sucrose overnight. Frozen sections (12 μm) were immunostained with rabbit anti-PGP9.5 (1:100) and Alexa Fluor 488-conjugated secondary antibody, mounted with DAPI, and imaged by confocal microscopy. For each condition, >100 neuronal somata with visible nuclei were counted to calculate the percentage of immunopositive neurons.

### 2.10. Determination of Bupivacaine by High-Performance Liquid Chromatography (HPLC)

Bupivacaine release from PLGA nanoparticles was monitored in serum over 3 days. At scheduled time points (days 1, 2 and 3), serum samples were centrifuged at 15,000 rpm for 10 min. The supernatant was withdrawn and replaced with an equal volume of PBS and 10 µL was injected into the column. High-performance liquid chromatography analysis (Agilent 1260 Infinity, Agilent Technologies, Santa Clara, CA, USA) was performed using an Aglient ZORBAX SB-C18 column (Agilent Technologies, Santa Clara, CA, USA) (4.6 mm, 250 mm, 5 u particle size) at 30 °C, under the moving phase consisting of acetonitrile: NaH_2_PO_4_/H_3_PO_4_ (pH = 3) at a ratio of 20:80, with detection at 230 nm and a flow rate of 1.0 mL/min for 25 min [20]. Seven bupivacaine solutions of known concentrations ranging from 0.03906 to 2.5 mg/L were used to generate a standard linear calibration curve (R^2^ > 0.99). The EE and DL of Bupi-NP were determined using an ultrafiltration centrifugation method. Briefly, an aliquot of the nanoparticle suspension was centrifuged to separate the free drug (in the supernatant) from the encapsulated drug. For total drug content, the nanoparticle suspension was completely disrupted using DMSO to extract the encapsulated bupivacaine. Both the supernatant (free drug, W_free) and the disrupted suspension (total drug, W_total) were filtered through a 0.22 μm membrane and analyzed by HPLC. The EE (%) and DL (%) were calculated according to the following equations:

EE (%) = (W_total − W_free)/W_total × 100%

DL (%) = (W_total − W_free)/W_nanoparticles × 100%

### 2.11. Detection of Tissue Injury and Drug Toxicity

Under isoflurane anesthesia, the DRG tissues were obtained from the rats that received nanoparticle injection or not. Tissues were dehydrated, embedded in paraffin, and sliced into sections with a thickness of 5 μm. Hematoxylin and eosin (H&E) staining was performed according to the standard instructions. Toluidine blue staining was also conducted for detection of neuron tissue injury, improving the sensitivity of routine histology examination results [21]. Several visual fields were observed under 40 × light microscopy. A total of 48 sections from 12 ganglia were examined (24 from the control group and 24 from the nanoparticle injection group). Images showing neuronal morphology and structure were photographed by Cellsens Standard Software (Version 4.5, Evident, Tokyo, Japan). Rats were anesthetized with sodium pentobarbital (40 mg/kg) and then perfused through the left ventricle with ice-cold phosphate-buffered saline (PBS) followed by 4% paraformaldehyde (PFA, Servicebio, Wuhan, China). The L3–L5 dorsal root ganglia (DRG) were collected, fixed in 4% paraformaldehyde for 1 h, and then dehydrated in 30% (*w*/*v*) sucrose for 24 h at 4 °C before they were embedded and frozen in OCT compound (Tissue-Tek, Sakura Finetek Japan Co., Ltd., Tokyo, Japan). Tissues were cut to 12 µm in a cryostat for immunofluorescence staining. After being incubated with 5% horse serum for 1 h, tissue sections were incubated at 4 °C overnight with primary antibodies against CD68 (Abcam, AB283654, 1:200, Cambridge, UK) and Iba1 (Oasis, OB-PGP049, 1:500, Hangzhou, Zhejiang, China). After being washed with PBS, the tissues were incubated with appropriate secondary antibodies (Abcam, AB150077, AB150188, 1:400, Cambridge, UK) for 1 h at room temperature. The slides were cover-slipped with fluorescent mounting medium (Sigma-Aldrich, F6057, St. Louis, MO, USA) and imaged after drying. Images were captured by a laser confocal microscopic imaging system (Olympus FV1000 and FluoView software, Ver. 4.2b, Olympus Corporation, Tokyo, Japan).

### 2.12. Statistical Analysis

Values were expressed as group mean ± standard error of the mean (mean ± SEM) and were analyzed using GraphPad Prism 6 and SPSS 19. Normality was assessed using the Shapiro–Wilk test, and homogeneity of variances was tested using Levene’s test. For behavioral data, two-way analysis of variance (ANOVA) was used to evaluate the effects of treatment and time, followed by Tukey’s post hoc test for multiple comparisons when a significant interaction or main effect was found. When the assumptions of ANOVA (normality or homoscedasticity) were violated, data were either log-transformed or analyzed using the non-parametric Kruskal–Wallis test with Dunn’s post hoc test. For categorical data, incidence rates between groups were compared using the chi-square test. In all analyses, a two-sided *p* < 0.05 was considered statistically significant. No adjustment for multiple comparisons was made beyond the planned post hoc tests. Sample size was determined based on an a priori power calculation.

## 3. Results

### 3.1. Nanoparticle Characterization

Under scanning electron microscopy, the bupivacaine-encapsulated PLGA nanoparticles appeared morphologically spherical with a mean diameter of approximately 150 ± 10 nm (Figure 1A). When fully dissolved in DMSO solvent, the weight of bupivacaine in each 2 mg of nanoparticle was 0.48 mg.

Bupivacaine showed great crystallinity as indicated by sharp peaks at 2θ of 9.78°, 20.04°, 21.36°, 23.76°, 25.348° and 26.64° (Figure 1B). The crystallinity of PLGA was denoted by a peak at 2θ of 19.02° (Figure 1B). Bupivacaine-loaded PLGA nanoparticles exhibited crystalline peaks at 2θ of 9.94° and 21.48°. To obtain indirect evidence for neuronal entry of bupivacaine nanoparticles, we co-encapsulated DiI as a tracer. After CCD, DRG sections were examined for DiI fluorescence, and immunostaining was used to confirm that the observed signal originated from nanoparticles inside neurons (Figure 1C–E). The Z-average size, PDI, and zeta potential were measured by dynamic light scattering and electrophoretic light scattering. Data are presented as mean ± SD (*n* = 3). Hydrodynamic diameter distribution of Bupi-NP was determined by dynamic light scattering (DLS). The *X*-axis represents the particle diameter (d, nm) on a logarithmic scale, and the *Y*-axis shows the intensity percentage. The average particle size is approximately 205.0 ± 3.5 nm (Figure 1F, Table 1). The drug loading (DL) and encapsulation efficiency (EE) of bupivacaine-loaded PLGA nanoparticles (Bupi-NP) were 22.17% and 98.96%, respectively (Table 1).

The characteristic absorption peaks of PLGA at ~1750 cm^−1^ (C=O stretching) and ~1100–1200 cm^−1^ (C–O–C stretching) are observed in both NP and Bupi-NP. The spectra confirm the successful incorporation of bupivacaine into the PLGA-based nanoparticles without significant chemical interactions (Figure 1G).

^1^H NMR (400 MHz, CDCl_3_): δ 7.28–7.14 (m, Ar-H, bupivacaine), 5.22–5.17 (m, PLA –CH–, PLGA), 4.82 (br s, PGA –CH_2_–, PLGA), 3.71 (d, J = 12.5 Hz, bupivacaine 6-CH), 2.41 (d, J = 14.1 Hz, bupivacaine 3-CH), 2.17 (s, Ar-CH_3_, bupivacaine), 1.55–1.58 (m, PLA –CH_3_, PLGA), 1.36 (h, J = 7.4 Hz, bupivacaine 9-CH_2_), 0.90 (t, J = 7.4 Hz, bupivacaine 10-CH_3_) (Figure 1H).

### 3.2. In Vivo Electrophysiological Recording

In vivo DRG electrophysiological recordings were used to measure spontaneous firing of neuronal somata and their responses to mechanical stimuli, as illustrated in Figure 2A–D. We recorded 54 neurons from 10 mice (*n* = 34 neurons from 5 mice in the CCD group, and *n* = 20 neurons from 5 mice in the CCD + Bupi group). After CCD, 32.4% (11/34) of neurons from five mice exhibited spontaneous activity (SA) (Figure 2E). Neurons in CCD + Bupi mice (*n* = 5) were silent, i.e., exhibited no SA. Action potentials (APs) were recorded from 34 neurons in five CCD mice and 20 neurons in five CCD + Bupi mice. Mechanical stimulation with von Frey filaments (tip 200 μm; forces 2–80 mN) evoked force-dependent increases in APs. CCD neurons had significantly higher mean AP counts across forces compared with CCD + Bupi (RM-ANOVA, treatment × force, *p* < 0.05); post hoc *t*-tests with Holm–Bonferroni correction confirmed significance at each force (Figure 2F).

The temporal pattern of discharge in mechanosensitive C-nociceptors conveys intensity, pain-like behavior, and hyperalgesia. Histograms of spontaneous (Figure 3A) and evoked (20–80 mN, Figure 3B–D) discharges were generated for CCD and CCD + Bupi neurons; evoked frequency distributions shifted rightward versus spontaneous in both groups.

### 3.3. Behavioral Studies in CCD Rats

No obvious signs of motor impairment from CCD were observed in rats. At baseline, there were no significant differences in the paw withdrawal mechanical threshold (PWMT) between the two treatment groups or between the values obtained for each paw (Figure 4). For the contralateral paw, there were no significant changes in PWMT for the two groups on postoperative days after CCD with bupivacaine nanoparticles or CCD with blank nanoparticles. For the ipsilateral paw, PWMT was significantly lower on each postoperative day after CCD with bupivacaine nanoparticles compared with baseline. The threshold of ipsilateral paw withdrawal to force was not significantly changed except on postoperative days 4 and 14 after blank nanoparticles.

For both CCD groups, postoperative PWMT was significantly lower on each postoperative day than that of the contralateral side, indicating that CCD induced mechanical hyperalgesia that persisted for at least 14 days. Furthermore, the PWMT was significantly lower at postoperative days 1, 4, 7 and 14 for the bupivacaine nanoparticle group compared to the blank nanoparticle group in response to mechanical stimulation. Thus, bupivacaine prevented the manifestation of CCD-induced hyperalgesia.

### 3.4. Behavioral Assessment and Electrophysiological Recordings in CCI Rats

Early analgesic treatment of pain can prevent the occurrence of chronic pain. In order to determine whether bupivacaine nanoparticles can produce a sustained analgesic effect on chronic pain rather than only early administration, we first established the chronic pain model of CCI rats (*n* = 6), and then injected bupivacaine nanoparticles around the ligated sciatic nerve on the 7th day after CCI operation. Our results show that bupivacaine nanoparticles exerted an analgesic effect on chronic mechanical pain from day 8 to day 28 after CCI (Figure 5A,B), but had no effect on pain caused by thermal stimulation.

We next examined the compound action potentials (CAPs) in the presence of bupivacaine nanoparticles. The sciatic nerve in rats was exposed and CAPs were evoked by electrical stimuli from the distal part and recorded extracellularly from the proximal part using a silver wire electrode.

Local application of capsaicin elicited electric impulses of both A-fibers and C-fibers in the sciatic nerve (Figure 5C). Notably, upon injection of 10 μL of bupivacaine nanoparticle suspension into the 1 mL small well, the transmission of nerve impulses in C-fibers was inhibited within a few seconds whereas activity of A-fibers was not affected. This formulation produced a relatively prolonged selective inhibition, as indicated by the absence of electric action potential in C-fibers lasting for at least 3 min (Figure 5D,E). Persistent inhibition ceased only after the suspension containing bupivacaine was washed out by replacement with an equal volume of saline solution. Then the impulse transduction in C-fibers immediately recovered (Figure 5F). 

### 3.5. Local Inflammation and Toxicity Study

We conducted H&E staining and toluidine blue staining to directly assess whether nanoparticle injections cause tissue injury in the ganglia. DRG harvested from naïve rats were used as negative controls (Figure 6A,C). Light microscopic images demonstrated that the neuronal cell bodies in DRG receiving nanoparticle injection were relatively intact with a staining profile similar to that of the control group. No significant changes in total cell numbers were observed in selected visual fields. Neither inflammatory infiltration nor histological damage was evident in either group, including cell edema, cell lysis, structure destruction, nuclear condensation, nuclear degradation, and organelle loss (Figure 6B,D) [22].

### 3.6. Blood Absorption Study

Blood samples were collected and examined on postoperative days 1, 3, and 5 (Figure 7). The plasma concentrations of bupivacaine that triggered convulsions and cardiac arrest in rats were 7.9 ± 1.2 and 21.6 ± 3.3 μg/mL, respectively [23].

The peak release occurred within 1 day in mice receiving nanoparticle injection and the drug concentration detected (156 ng/mL) was much lower than the theoretical toxicity amount, calculated based on blood volume in the rat. Release of bupivacaine in mice was continuous and stable on the following days as reflected by the data from postoperative days 3 and 5. For the release kinetics of bupivacaine in rats, it differed slightly from that in mice: the serum drug concentration reached a maximum on postoperative day 3 rather than day 1. For the safety profile, serum bupivacaine levels detected in rats were similarly far below the toxicity threshold as mice (18 ng/mL). The in vivo release period of the PLGA nanoparticles was completed by approximately 120 h in both animal models, which was at least 15 times longer than that provided by free drug intravenous injection (6–8 h). 

### 3.7. Effect of Bupi-NP on Microglia/Macrophage Activation in the DRG of CCI Rats

To evaluate the effect of bupivacaine-loaded nanoparticles (Bupi-NPs) on neuroinflammation in the dorsal root ganglia (DRG), immunofluorescence staining was performed on L3–L5 DRG sections from CCI and CCI + Bupi groups. As shown in Figure 8A, the expression and co-localization of CD68 (a marker for activated microglia/macrophages, green) and Iba1 (a general microglia/macrophage marker, red) were assessed.

Quantitative analysis of the co-localized signals (CD68^+^/Iba1^+^) is presented in Figure 8B. The proportion of CD68^+^/Iba1^+^ cells in the CCI group (873/3140, ~27.8%) was significantly higher than that in the CCI + Bupi group (268/2928, ~9.1%) (**** *p* < 0.0001). These results indicate that Bupi-NP treatment significantly inhibited the activation phenotype of microglia/macrophages in the DRG of CCI rats.

## 4. Discussion

Our study demonstrated the efficacy and safety of bupivacaine-encapsulated PLGA nanoparticles for direct local injection into the DRG to produce sustained analgesia. Nanoparticles containing bupivacaine prevented the manifestation of mechanical hyperalgesia induced by CCD for at least 14 days. Electrophysiological recordings from sciatic nerves suggested that bupivacaine may act primarily by blocking the conduction activity of C-fibers innervating the peripheral receptive field, thereby verifying its function in the inhibition of chronic neuropathic pain. Study limitations include potential bias (e.g., lack of blinding, single-observer scoring) and imprecision due to small sample size (*n* = 5 or 6 per group). These factors may affect the reliability of our results.

Local anesthesia in peripheral nerve block provides a limited duration of no more than 24 h with single-dose injection [24]. Exparrel^®^, the only commercially available extended-release formulation, is composed of a liposome matrix and is currently applied in surgical infiltration and interscalene brachial plexus block [25,26]. It has been shown that liposome bupivacaine has the capacity to attenuate hyperalgesia for up to 72 h in humans when compared with bupivacaine hydrochloride or placebo [9]. In a rat model of incisional pain, local infiltration of liposome bupivacaine at a dose of 1 mg/kg effectively prevented the onset of mechanical and thermal hypersensitivity for 4 days [27]. In naïve rats, injection of liposome bupivacaine posteromedial to the great trochanter produced a 4 h median duration of sensory blockade of the sciatic nerve [28]. Other types of extended-release formulation currently under active development, such as sucrose acetate isobutyrate, have a similar duration to liposomal bupivacaine, in which sustained drug delivery was detected for 48–72 h after the drug was locally infiltrated in patients undergoing open inguinal hernia repair [29]. A designed bupivacaine-loaded cerium metal–organic framework (Ce-MOF) integrates sustained analgesia, immunomodulation, and antioxidant effects; in CCI models, it reduced neuroinflammation, promoted M2 microglial polarization, and enhanced myelin regeneration for prolonged analgesia [30]. An alternative approach was to encapsulate bupivacaine into PLGA nanoparticles as used in our study, which may offer a significant improvement over current strategies of analgesia extension, as evidenced by experimental results including continuous drug release over 35 days in vitro [12], sustained release over 5 days in rodent models and successfully elevating pain threshold for at least 14 days in CCD rats. These promising preclinical results attest to a need for further clinical trials to determine whether the analgesic duration and effect of this product applied in our animal models would be comparable to those in human subjects, i.e., what degree of clinical translation could be achieved.

A biodegradable poly(fumarate urethane) (PFU) for bupivacaine delivery degrades via ester bond hydrolysis/enzymolysis; a 2.5% drug loading gives the highest encapsulation and sustained release [31]. A thermosensitive gel with bupivacaine multivesicular liposomes and meloxicam nanocrystals adapts to acidic microenvironment, reduces IL-6/TNF-α, and provides ≥5-day analgesia [32]. Researchers developed a CS-GP/PC hydrogel (CS-GP shell, PC nanocapsule core) for bupivacaine, prolonging anesthesia/analgesia, with good biodegradability, biocompatibility, and no neurotoxicity [33]. Importantly, the approach described herein—direct injection of bupivacaine-encapsulated PLGA nanoparticles—appears to be safe and promising for future translational medicine. First, as for the release pharmacokinetics, PLGA nanoparticles elicited only a single peak compared to liposome bupivacaine in which an initial peak occurred within 1 h after drug administration, followed by a second peak 12–36 h later [34]. Thus, drug release from these nanoparticles is more easily controlled than that from competing nanocarrier systems, such as liposomes or micelles, probably lessening the risk of severe adverse tissue reactions. Second, PLGA is a polyester of biocompatibility and biodegradability which can be fully degraded into endogenous metabolite monomers via hydrolysis and eventually into CO_2_ and H_2_O via the Krebs cycle, minimizing the potential biotoxicity to a minimal extent [16]. Third, it bears mentioning that the drug delivery method itself causes no deleterious effects on the experimental animals, since the overall structure and function of injected ganglia were maintained intact and all treated rodents normally lived through the whole course of follow-up without any indications of motor impairment or accidental death. In short, this is a highly localized delivery platform adjuvant with a long-lasting formulation of local anesthetics contributing to the advances in selective pain medicine and a reduction in unwanted systemic effects. Regarding the significant difference between rats and mice, this may be explained by species-specific physiological and anatomical differences at the injection site, including tissue vascularity, lymphatic drainage, local pH and temperature, metabolic capacity, and the intensity of the inflammatory/fibrotic response to PLGA. Differences in body weight and injection volume relative to tissue mass may also contribute. Importantly, although the absolute values differed between species, the overall release/absorption trend was consistent: an initial burst phase followed by a sustained phase. Regarding the low amount on days 3 and 5, it should be emphasized that Figure 7 shows the bupivacaine concentration in the blood, not the local drug concentration at the target site. Because the formulation was administered locally to the injured tissue/at the site of nerve injury, the local drug concentration at the target site would be substantially higher than the systemic blood concentration. Therefore, the relatively low blood concentrations on days 3 and 5 do not necessarily indicate that the therapeutic dose is insufficient. Instead, they may reflect slow systemic absorption and effective local retention of bupivacaine. This is consistent with the sustained-release characteristics of PLGA–Bupi nanoparticles: after the initial burst release, the remaining drug is gradually released from the PLGA matrix as the polymer degrades. In addition, our in vivo behavioral results showed that analgesia was still maintained on days 3 and 5 (Figure 4 and Figure 5), supporting that the local drug exposure was therapeutically adequate. Moreover, the low systemic blood concentration is advantageous for reducing the risk of systemic toxicity and motor blockade.

The differential effect of bupivacaine-loaded PLGA nanoparticles on mechanical and thermal pain is consistent with the growing evidence that these two pain modalities are mediated by distinct neuronal mechanisms and are differentially sensitive to pharmacological intervention. First, clinical studies have demonstrated that local anesthetics produce modality-specific blockade. Brennum et al. showed that epidural bupivacaine in humans produced a modality-related blocking order for prolonged painful stimuli: heat > mechanical > electrical. This indicates that bupivacaine does not affect all pain modalities equally, and that thermal and mechanical nociception can be differentially modulated even by the same agent [35].

Second, in animal models of neuropathic pain, mechanical allodynia and thermal hyperalgesia have been shown to be pharmacologically dissociable. A study comparing multiple analgesics in CCI and SNL rats reported that mechanical allodynia produced by either injury showed “a noticeable difference in pharmacological sensitivity compared to heat hyperalgesia” [36]. Furthermore, genetic studies in different rat and mouse strains revealed no correlation between hypersensitivity to heat and von Frey mechanical stimuli following peripheral neuropathy, supporting the idea that heat and punctate mechanical hypersensitivity are mediated by distinct cellular mechanisms. Another study on the sodium channel blocker zonisamide similarly concluded that “the pathogenetic mechanisms responsible for thermal hyperalgesia and mechanical allodynia are probably not the same, either in the rat CCI model or in human neuropathic pain” [37]. 

The most likely mechanistic explanation for our findings is that the sustained, low-dose release of bupivacaine from PLGA nanoparticles preferentially suppresses the abnormal mechanical hypersensitivity that arises from ectopic firing and central sensitization in the injured nerve or DRG, while the thermal nociceptive pathway—particularly the heat-evoked withdrawal response mediated by TRPV1-positive C-fibers—may require a higher local concentration of sodium channel blockade to be suppressed, or may be less dependent on the specific sodium channel subtypes (e.g., Nav1.7, Nav1.8) that are effectively modulated by the slow-release profile achieved with our nanoparticle formulation.

PLGA nanoparticles can greatly enhance the efficacy of bupivacaine with reduced toxicity in preventing or controlling chronic neuropathic pain. A long and steady release profile was observed in this nanoformulation, potentially offering a significant increase on current nanodrug delivery system.

## 5. Conclusions

In summary, bupivacaine-encapsulated PLGA nanoparticles, administered via direct local injection, produced sustained analgesia lasting at least 14 days in rodent models of chronic neuropathic pain. The analgesic effect was specific to mechanical hyperalgesia, with no impact on thermal pain, likely due to selective inhibition of C-fiber conduction. Plasma bupivacaine levels remained far below toxic thresholds, and no tissue damage was observed at the injection site. Compared with existing extended-release formulations, our PLGA-based system offers longer duration and a simpler release profile, supporting its potential as a safe and effective alternative for chronic pain management.

## Figures and Tables

**Figure 1:**
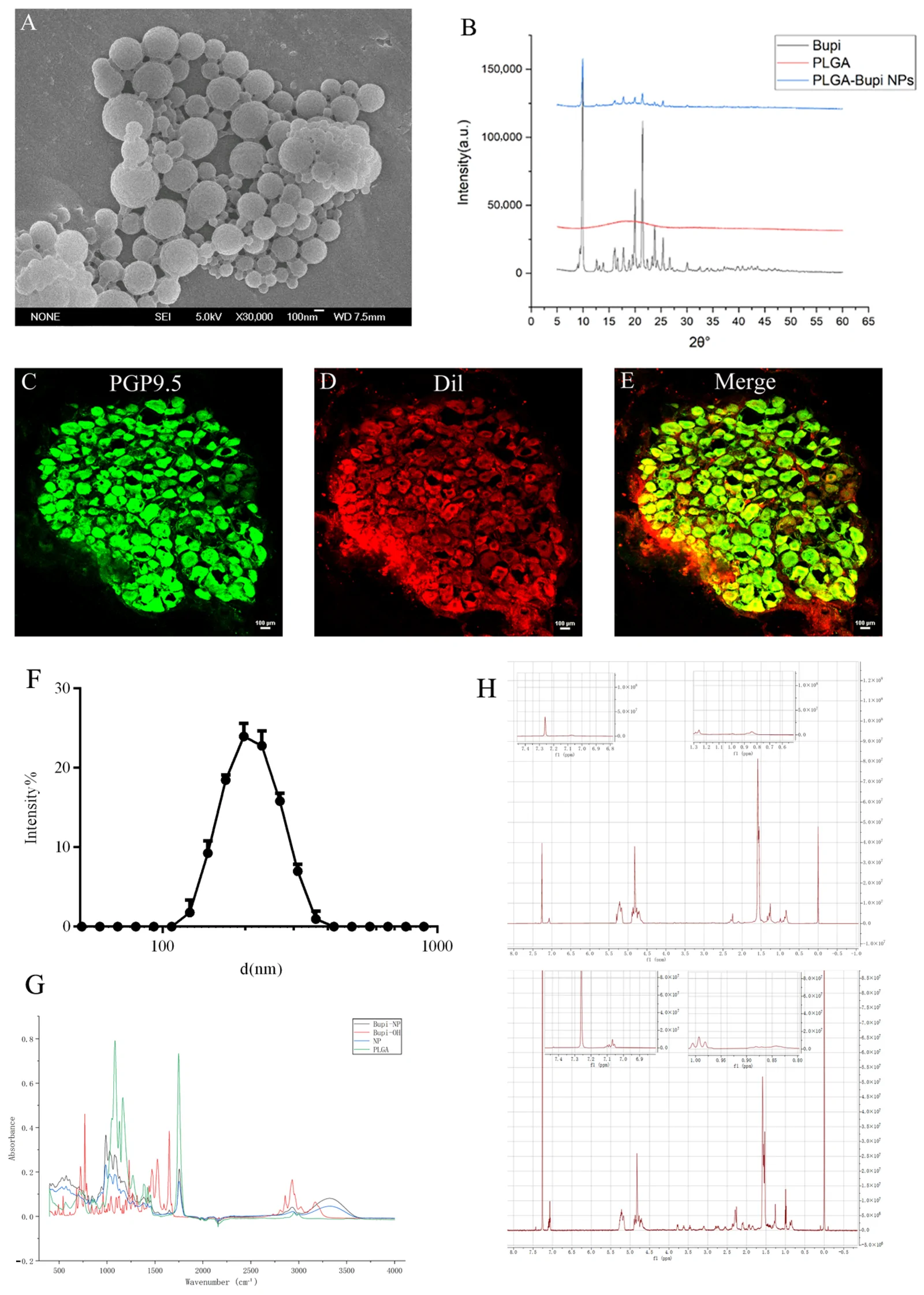
(**A**) Morphology of bupivacaine-encapsulated PLGA nanoparticles with a mean diameter of 150 ± 10 nm under scanning electron microscopy (scale bar = 1 μm). (**B**) X-ray diffraction (XRD) pattern obtained from powder form of bupivacaine, crystalline form of PLGA and bupivacaine-loaded nanoparticles. (**C**–**E**) Fluorescent images of neurons labeled with DiI (red) and immunostained with anti-PGP9.5 (green) in the L4 DRG 7 days after CCD surgery (scale bar = 100 μm). (**F**) Particle size distribution of PLGA–Bupi NPs was determined by DLS; the *y*-axis represents the relative scattering intensity (%) as a function of hydrodynamic diameter (nm). (**G**) FTIR spectra of PLGA (green), NP (blue), Bupi-OH (red), and Bupi-NP (black). (**H**) ^1^H NMR (400 MHz, CDCl_3_) of PLGA-bupivacaine. Insets: expansions of the aromatic (7.4–6.8 ppm) and high-field (1.0–0.8 ppm) regions.

**Figure 2:**
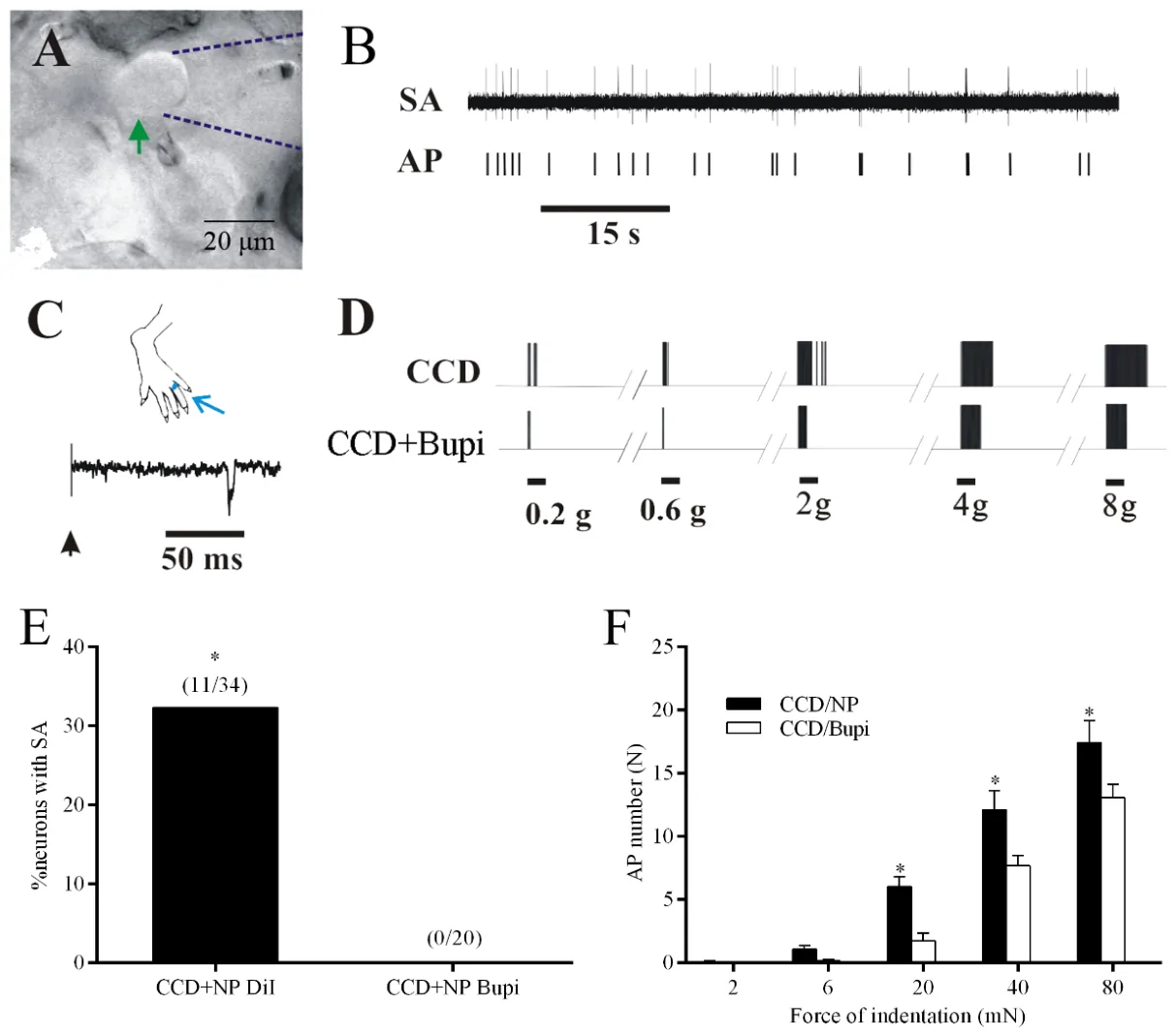
Spontaneous and mechanically evoked action potential activity electrophysiologically recorded in vivo from cell bodies of C-nociceptors. (**A**) Bright-field image of the cell body of a neuron whose action potentials are extracellularly recorded via a glass micropipette (outlined by dashed dark blue lines) in the DRG of a CCD mouse. (**B**) Spontaneous activity of a C-mechanosensitive nociceptor (CM) recorded after CCD (receptive field on finger, CV = 0.6 m/s). (**C**) The neuron was identified as a CM with a receptive field (RF) on the dorsum of the hairy skin (blue dot) that was electrically stimulated (Black arrow, start of electrophysiological recording; blue arrow, receptive field for mechanical stimulation.) to obtain its conduction velocity (CV) (0.6 m/s). (**D**) Responses of two C-mechanosensitive nociceptive neurons to von Frey filaments each with the same tip diameter of 200 μm but delivering different bending forces of 2, 6, 20, 40, and 80 mN. Two neurons recorded from a CCD mouse and a CCD Bupi mouse are shown. (**E**) Spontaneous activity of neurons from CCD mice and CCD Bupi mice. The single asterisk indicates a significant difference of *p* < 0.05. (**F**) Mean number of action potentials evoked in neurons from CCD Bupi mice (open bars) and CCD mice (closed bars).

**Figure 3:**
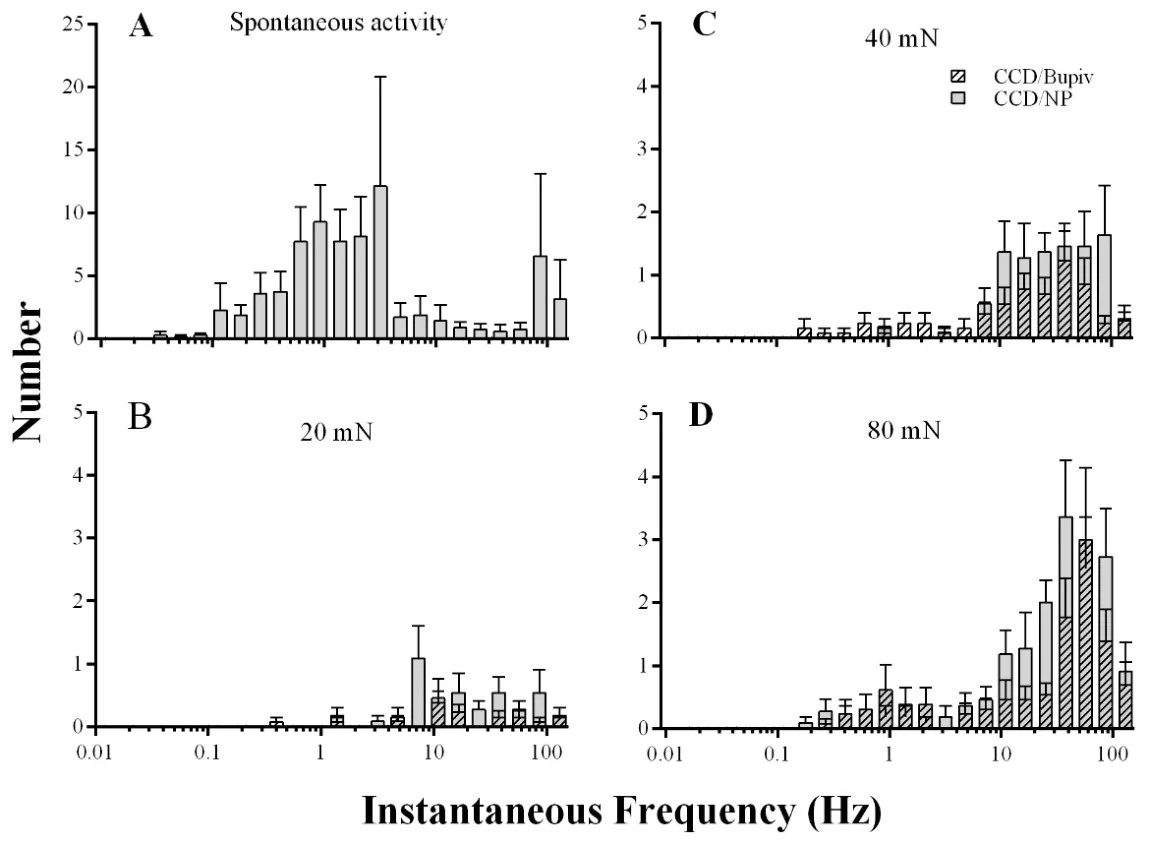
Histograms of instantaneous frequencies of spontaneous and mechanically evoked responses of C-mechanosensitive nociceptors. The number of instantaneous frequencies that occurred within each bin (log increments, factor $\sqrt{2}$) is combined across nociceptors and plotted for CCD mice (closed bars) and CCD Bupi mice (hatched bars). (**A**) Spontaneous activity, observed only after CCD. (**B**–**D**) Responses to 20, 40 and 80 mN forces delivered by von Frey filaments each with the same tip diameter of 200 μm.

**Figure 4:**
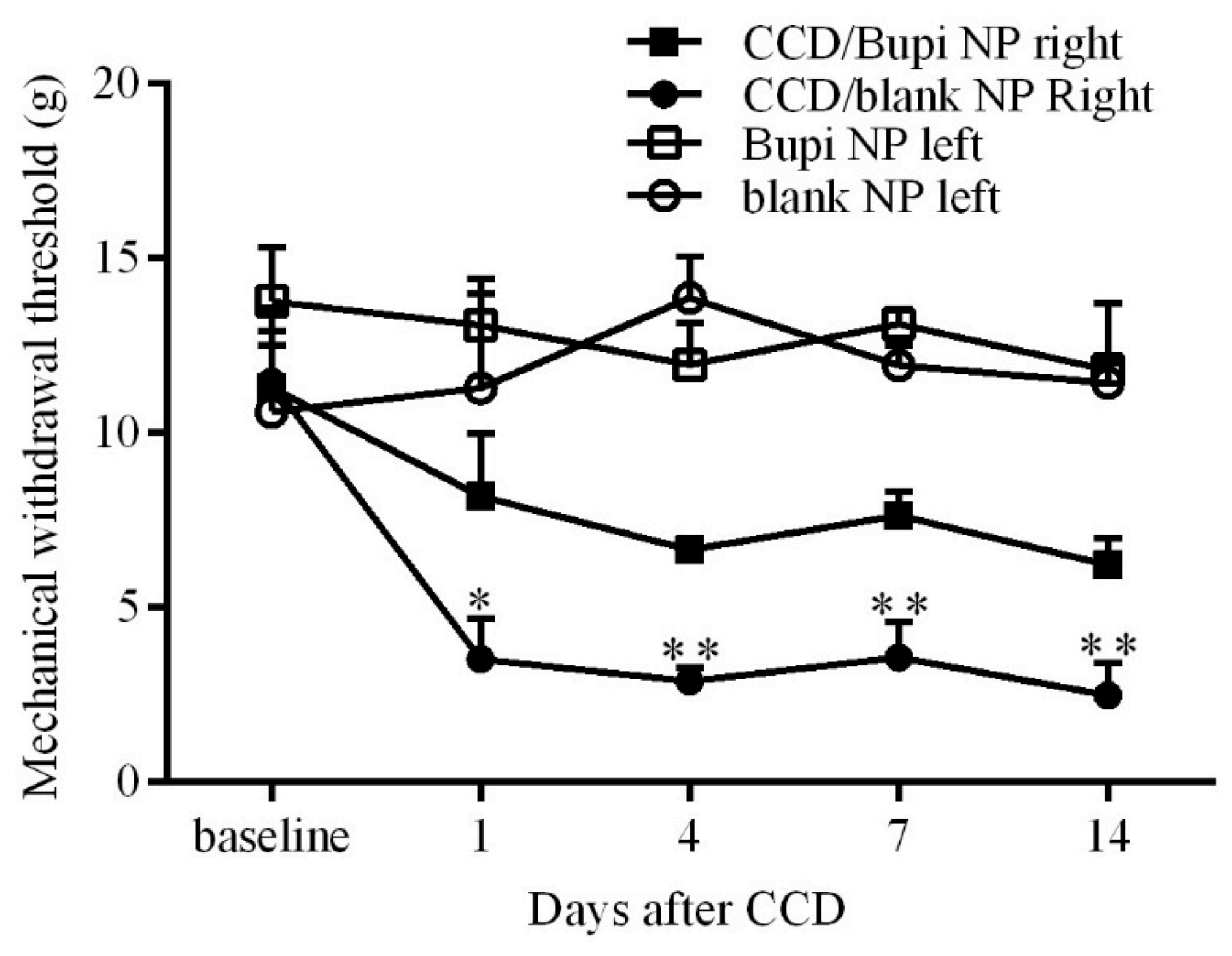
Behavioral assessment in a chronic compression of the dorsal root ganglion model in rats to evaluate the mechanical pain withdrawal threshold (MPWT) of each paw to von Frey filaments on different postoperative days. Each point indicates the mean value (±SEM) in MPWT. Rats receiving CCD and bupivacaine nanoparticles (solid squares) or CCD with blank nanoparticles (no drug) (solid circles) were tested. The contralateral paws of CCD are shown by open squares (with bupivacaine nanoparticles) or open circles (with blank nanoparticles). The double asterisks indicate significant differences (*p* < 0.01) in MPWT between rats receiving bupivacaine nanoparticles vs. blank nanoparticles tested on the paw ipsilateral to the CCD, while the single asterisk indicates a significant difference of *p* < 0.05. SEMs not shown are similar to those displayed.

**Figure 5:**
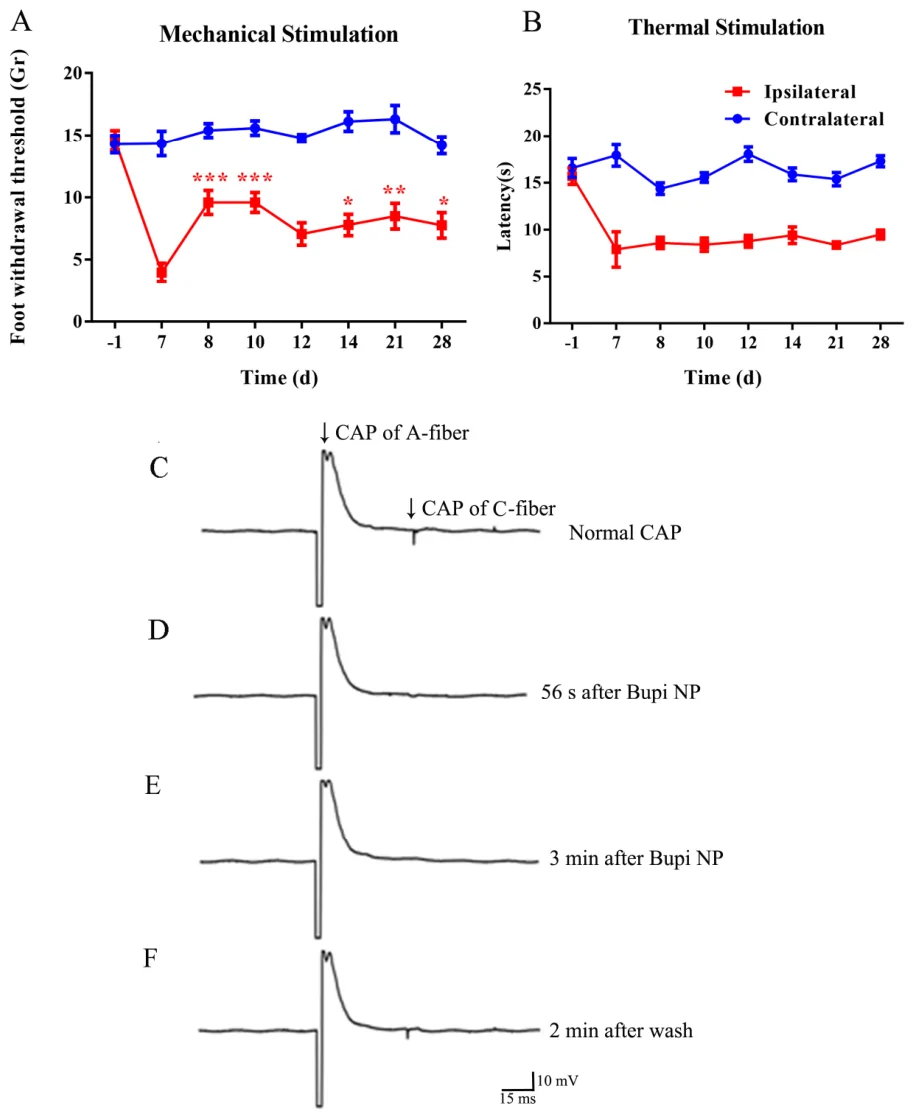
Behavioral detection of chronic constriction injury (CCI) model in rats. Each point indicates the mean value (±SEM) of paw withdrawal mechanical threshold (PWMT) in (**A**) and paw withdrawal thermal latency (PWTL) in (**B**). Rats receiving CCI with bupivacaine nanoparticles (ipsilateral, solid squares, and contralateral, solid circles) were tested. Behavioral comparison was made between 8 and 28 days after perineural injection of bupivacaine nanoparticles (administered on day 7 post-CCI) and baseline (day 7 post-CCI). *, *p* < 0.05; **, *p* < 0.01; ***, *p* < 0.001. Bupivacaine nanoparticles blocked the impulse conduction of C-fibers selectively without impairing the conduction capacity of A-fibers. (**C**) Capsaicin injection induced compound action potential in both A-fibers and C-fibers of sciatic nerve before application of nanoparticle suspension. (**D**,**E**) Nanoparticle injection caused immediate disappearance of compound action potential in C-fibers while activity of A-fibers was preserved. (**F**) 2 min after elution of physiological saline solution, recurrent electric impulse was recorded in C-fibers.

**Figure 6:**
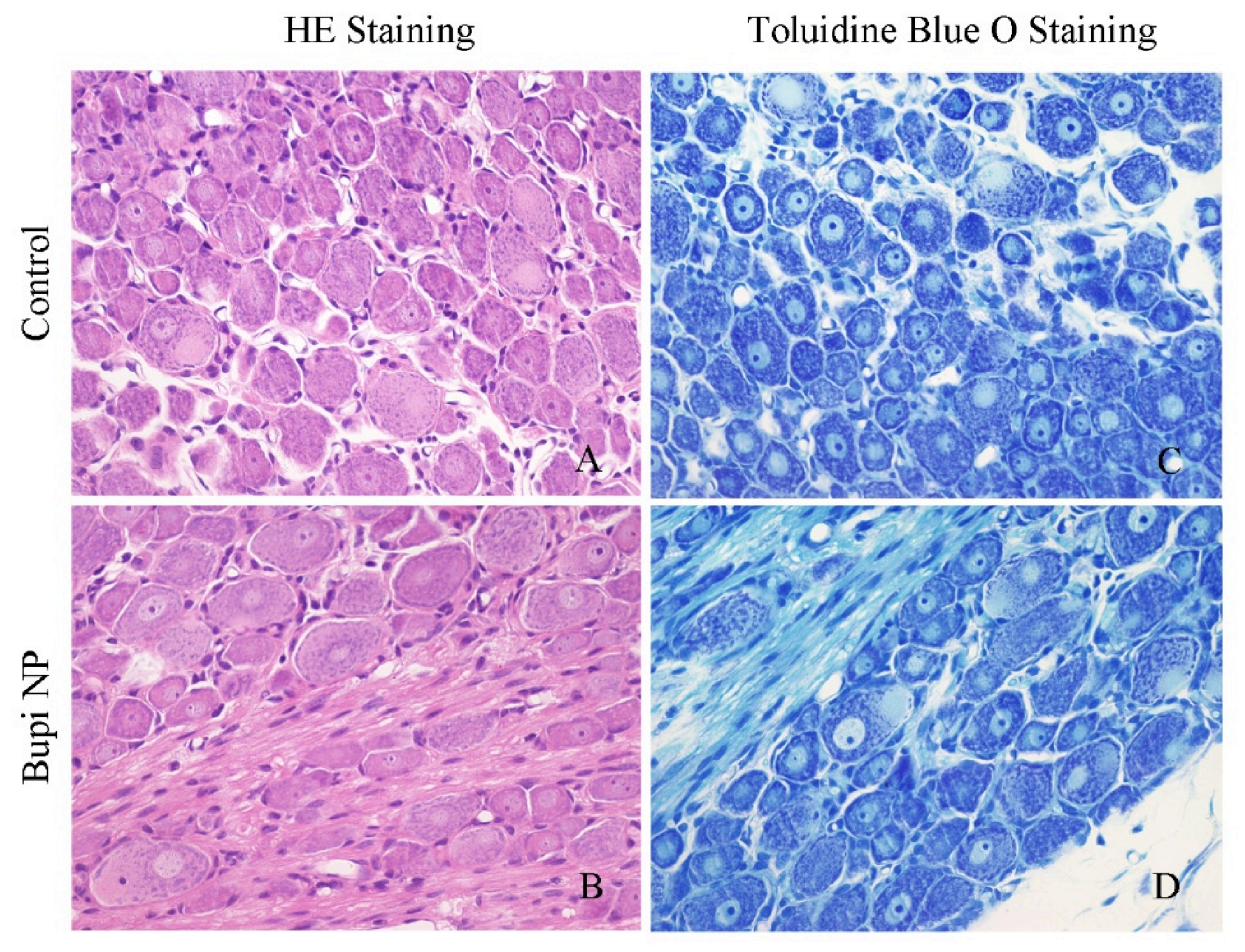
Assessment of neuron injury after injection of nanoparticle suspension in rats. DRG tissues with or without nanoparticle injection were subjected to histological tests including HE staining and toluidine blue staining. (**A**–**D**) Images are presented at 40× magnification.

**Figure 7:**
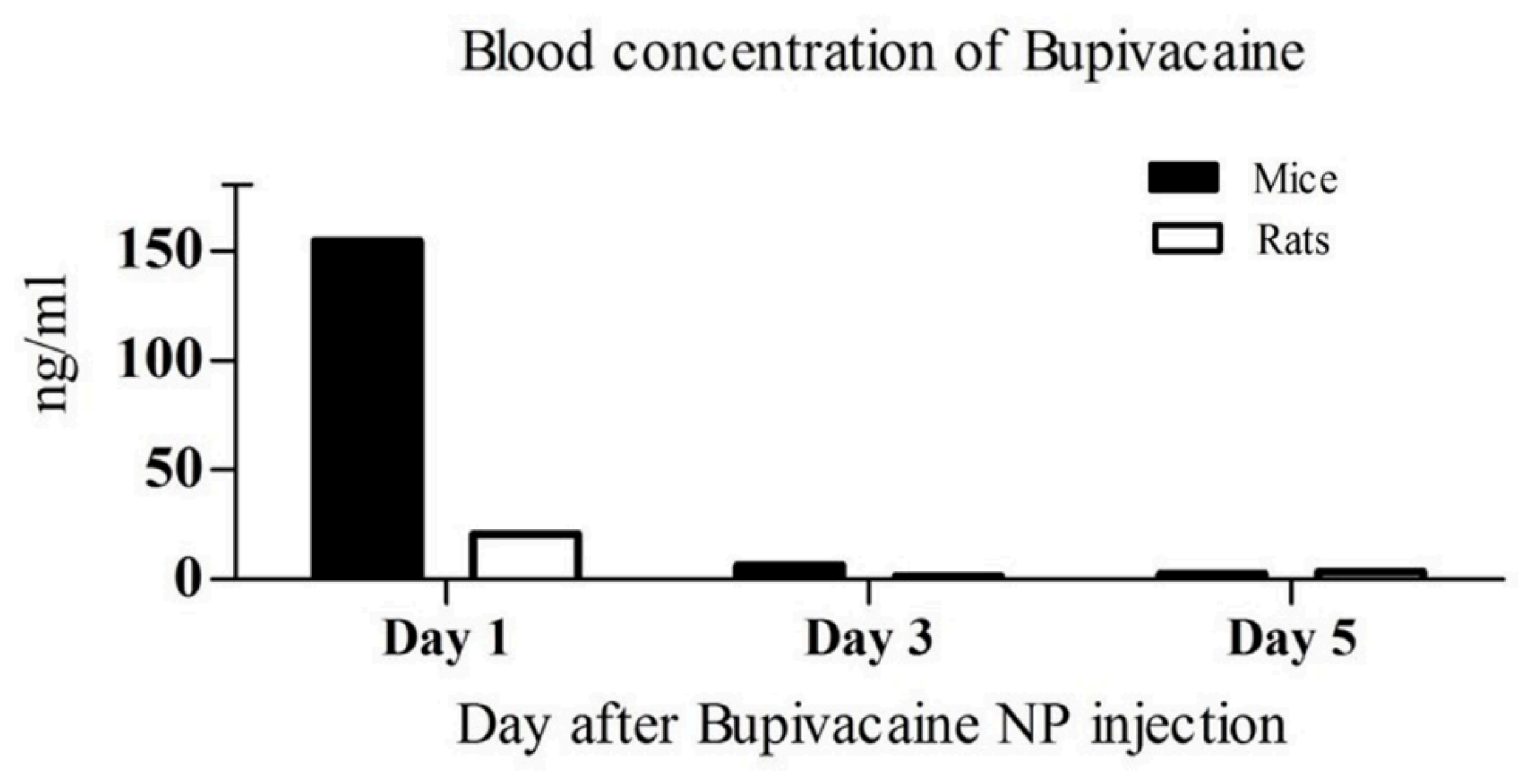
Assessment of pharmacokinetic characteristics of bupivacaine nanoparticles in rats and mice. Serum bupivacaine levels were recorded on postoperative days 1, 3 and 5, showing a single peak of release that remained below the minimal toxicity threshold. Data are presented as mean ± SD. Black bar: mice (*n* = 5); white bar: rats (*n* = 5).

**Figure 8:**
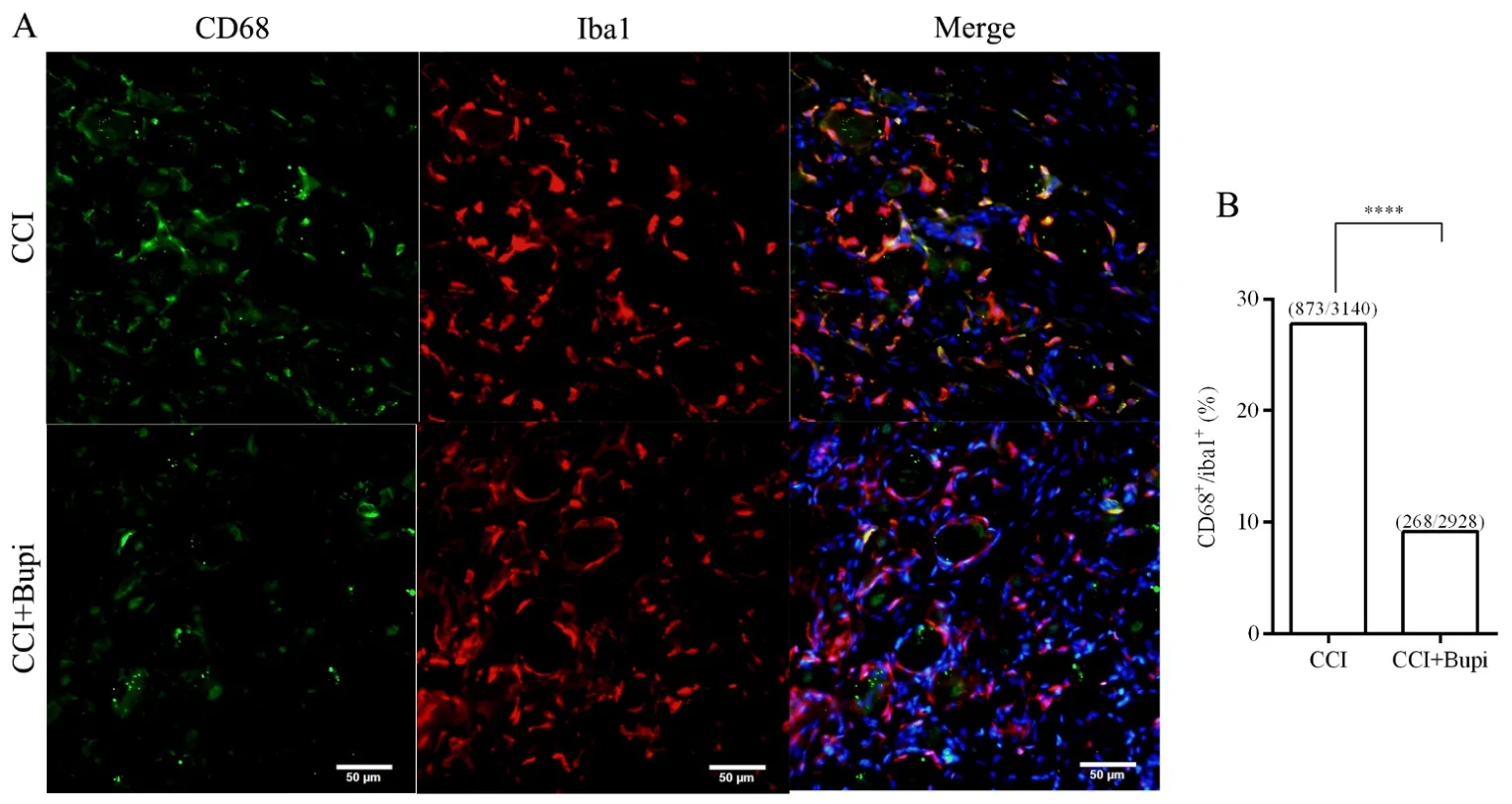
Immunofluorescence staining of CD68 and Iba1 in the L3–L5 DRG of CCI and CCI + Bupi rats. (**A**) Representative immunofluorescence images showing the co-localization of CD68 (green) and Iba1 (red) in the L3–L5 DRG. Nuclei were counterstained with DAPI (blue). Scale bars: 50 μm. (**B**) Quantitative analysis of the percentage of CD68^+^/Iba1^+^ cells. The numbers in parentheses represent the absolute cell counts (CD68^+^/Iba1^+^ cells/total Iba1^+^ cells). Data are presented as mean ± SD (*n* = 3 per group). **** *p* < 0.0001 vs. the CCI group.

**Table 1:** Physicochemical characterization of blank PLGA nanoparticles (NP) and bupivacaine-loaded PLGA nanoparticles (Bupi-NP). The Z-average size, PDI, and zeta potential were measured by dynamic light scattering and electrophoretic light scattering. Data are presented as mean ± SD (*n* = 3).

Figure.	Z-Average Size (nm)	PDI	Zeta Potential (mV)	EE (%)	DL (%)
NP	247.2 ± 11.8	0.037 ± 0.014	−15.6 ± 1.6		
Bupi-NP	205.0 ± 3.5	0.018 ± 0.016	−10.7 ± 1.3	98.96	22.17

Data are presented as mean ± SD (*n* = 3). PDI, polydispersity index.

## Data Availability

All data are available in the manuscript.
